# Ulcer of the Stomach Treated with Protonuclein

**Published:** 1896-10

**Authors:** H. N. Kirby

**Affiliations:** Concord, Mass.


### ULCER OF THE STOMACH TREATED WITH PROTONUCLEIN.

By H. N. KIRBY, M. D., Concord, Mass.

MY EXPERIENCE with protonuclein has been limited, but in all cases in which I have used it I have been quite successful, especially so in the one which is reported below:

Mr. J., aged 24, farmer by occupation, became one of my patients some two years ago, when he presented the following symptoms: For a long time he had been troubled with inability to retain food. There would be severe pain in the stomach, which would be increased by the presence of food and by pressure over region of stomach. This pain would be relieved by vomiting; sometimes there would be vomiting of blood; bowels constipated, tongue covered with thick coating. I tried several remedies at that time, such as pepsin, bismuth, nitrate of silver, aromatic powder, Carlsbad salts, mustard over epigastrium, and the like, together with a strict diet. This course of treatment was followed by temporary improvement, but no real improvement. He finally left me, utterly discouraged, and came under the care of other physicians, but with apparently no better success. About two months ago he returned to me, very much reduced in flesh, his weight having dropped from 160, his normal weight at the beginning of the trouble, to 124 pounds, and every symptom increased in its severity, and so weak and exhausted was he that he was unable to follow his usual occupation.

I straightway put him upon a strict diet again, and gave him protonuclein, grs. iii., every four hours, to be taken religiously. From the beginning of the administration of protonuclein he began to improve and gradually to retain food. The pain began to diminish and he gained fully ten pounds in weight within the first two weeks. His appetite and strength returned, and in fact there was rapid and permanent improvement. At the present time of writing he has ceased taking protonuclein, and when I last saw him he was apparently well.—*Atlanta Medical Weekly*.

